# Evolution of the vertebrate skeleton: morphology, embryology, and development

**DOI:** 10.1186/s40851-014-0007-7

**Published:** 2015-01-13

**Authors:** Tatsuya Hirasawa, Shigeru Kuratani

**Affiliations:** Evolutionary Morphology Laboratory, RIKEN, 2-2-3 Minatojima-minami, Chuo-ku, Kobe, Hyogo 650-0047 Japan

**Keywords:** Vertebrate, Skeleton, Evolution, Development, Homology

## Abstract

Two major skeletal systems—the endoskeleton and exoskeleton—are recognized in vertebrate evolution. Here, we propose that these two systems are distinguished primarily by their relative positions, not by differences in embryonic histogenesis or cell lineage of origin. Comparative embryologic analyses have shown that both types of skeleton have changed their mode of histogenesis during evolution. Although exoskeletons were thought to arise exclusively from the neural crest, recent experiments in teleosts have shown that exoskeletons in the trunk are mesodermal in origin. The enameloid and dentine-coated postcranial exoskeleton seen in many vertebrates does not appear to represent an ancestral condition, as previously hypothesized, but rather a derived condition, in which the enameloid and dentine tissues became accreted to bones. Recent data from placoderm fossils are compatible with this scenario. In contrast, the skull contains neural crest-derived bones in its rostral part. Recent developmental studies suggest that the boundary between neural crest- and mesoderm-derived bones may not be consistent throughout evolution. Rather, the relative positions of bony elements may be conserved, and homologies of bony elements have been retained, with opportunistic changes in the mechanisms and cell lineages of development.

## Introduction

*“Is histological development as complete a test of homology as morphological development?” (Huxley, 1864* [1]*: 296)*

The vertebrate skeletal system has paramount importance for analyses in evolutionary biology. Because vertebrate skeletons can be viewed as aggregates of apparently discrete units, namely bones, they have attracted the interest of comparative anatomists since even before the dawn of the concept of evolution [2]. In addition, because bones can be preserved as fossils, comparative research can include extinct vertebrates, thereby shedding light on evolutionary patterns and processes (e.g., [3]). In addition, the vertebrate skeletal system is well suited to biomechanical analyses, allowing both morphological and functional transitions throughout evolution to be reconstructed (e.g., [4]).

In any comparative study, homology is a conceptual basis for comparing equivalent units. There is, however, a difficulty in establishing homology—that is, “the apparent loose relationship between morphological characters and their genetic basis” [5]. Incongruities between morphologies and their genetic bases may lead to errors when homology is defined solely according to criteria of ontogeny.

Skeletal systems of vertebrates are intolerant of such incongruities (reviewed by [6]). Historical continuities of skeletal elements as step-wise morphological changes along a phylogenic lineage are inferable from detailed comparative analyses. However, within these continuities, discontinuities of genetic and developmental bases arise in which morphologically homologous bones are produced through different developmental processes [7,8].

Before the concept of evolution was established, two distinct types of bones were recognized in vertebrate skeletons and were thought to reflect their embryonic development; specifically, whether the bone arose from a cartilaginous precursor or not (e.g., [9,10]). Bone arising from precursor cartilage develops not only on the surface of the cartilage (perichondral ossification), but also within the cartilage mass as the cartilage template becomes degraded (endochondral ossification), thereby distinguishing this type of bone from that lacking a cartilaginous precursor in terms of developmental process, or histogenesis. This line of demarcation in histogenesis was later considered to reflect the evolutionary succession of bones. For example, Huxley (1864: 298) [1] wrote, “It is highly probable that, throughout the vertebrate series, certain bones are always, in origin, cartilage bone, while certain others are always, in origin, membrane bone.” In addition, differences in the cell type of the osteoblast precursors—either mesodermal or neural crest cells—has historically been offered in support of the notion that these two histogenetically distinct types of bone generally evolved separately. However, here, we confirm, through a review of both classical and recent research, that both histogenesis and cell lineage are decoupled with the two independent lineages of skeletal systems, namely endo- and exoskeletons, the continuities of which are inferable from comparative morphology.

In this review, we first summarize various evolutionary continuities of vertebrate skeletal systems. We then describe their developmental bases at two hierarchal levels, namely histogenesis and cell lineage, according to recent studies in developmental biology. In light of this understanding, we discuss the loose relationship between morphology and developmental basis and suggest that a frame shift in character identity occurred across cell lineages during the evolution of vertebrate skeletal systems.

### Morphological divisions—endoskeleton vs. exoskeleton

**Figure 1:**
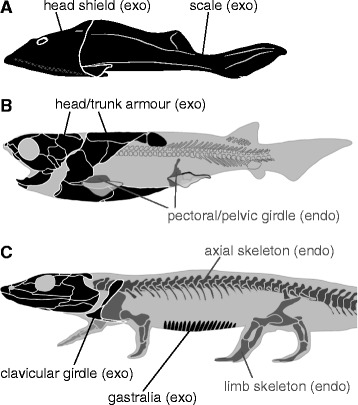
**Distribution of endoskeletons (endo) and exoskeletons (exo) in the vertebrate body.** **(A)** Osteostracan *Cephalaspis* (redrawn from [13]). **(B)** Basal jawed vertebrate *Compagopiscis* (redrawn from [14]). **(C)** Temnospondyl tetrapod *Dendrerpeton* (redrawn from [15]).

From the perspective of comparative morphology, including paleontology, it has been suggested that two lineages of skeletal systems—the endoskeleton and exoskeleton—have succeeded in vertebrate evolution (Figure 1, Table 1) [7,11]. This mode of classification is defined exclusively by phylogenetic continuities, and thus differs from terminology based on ontogeny [7]. For example, the endoskeleton consists of bones preformed from cartilage and their evolutionary derivatives, or homologues (Table 1) [7]. Most endoskeletal bones, such as those in the axial and limb skeletons, are located together with muscles within a deep layer of the body. However, in the evolution toward turtles, the thoracic axial skeleton was exposed, owing to loss of the dorsal axial muscles, to form the carapace [12]. In this sense, the turtle carapace should be considered an “exposed endoskeleton.” The distal tip of the distal (ungual) phalange is another example of an exposed endoskeleton that is recognized in vertebrates [12].

**Table 1:** **Classification of skeletal systems**

**Skeletal system**	**Bone**	**Example**
Endoskeleton	Cartilage bone	Vertebrae, ribs, limb bones
	Membrane bone	Centra of teleosts, sesamoid, orbitosphenoid of the Amphisbaenia
Exoskeleton	Dermal bone	Skull roof bones, dentary, clavicle, gastralia, scale of fishes, osteoderm

There is convincing evidence that cartilaginously preformed bones changed during evolution to become intramembranous bones. For example, the orbitosphenoid, a cranial skeletal element, of the Amphisbaenia (Reptilia: Squamata) develops intramembranously, although it clearly is homologous with the cartilaginously preformed orbitosphenoid of other tetrapods [16]. Patterson (1977) [7] proposed calling such intramembranous bones “membrane bones” and discriminated them from bones that developed within the dermis, or “dermal bone.” According Patterson’s terminology, the endoskeleton consists of cartilage and membrane bones (Table 1: Note that the above-mentioned Huxley’s definition of “membrane bone” is different from that used in this paper, as he did not distinguish dermal bones from other intramembranously formed bones).

In contrast, the exoskeleton consists of dermal bones (*sensu* [7]), which are homologous with bony armor and are often coated with enameloid or dentine tissues in basal vertebrates (Figure 1, Table 1; [17]). Exoskeletal bones are located superficially in the body in ancestral conditions, but some exoskeletal bones, such as the dentary and clavicle of mammals, have shifted in their positions to a layer deeper than that of some muscles [18-20]. In this sense, the dentary and clavicle might be referred to as “sunken exoskeleton.”

**Figure 2:**
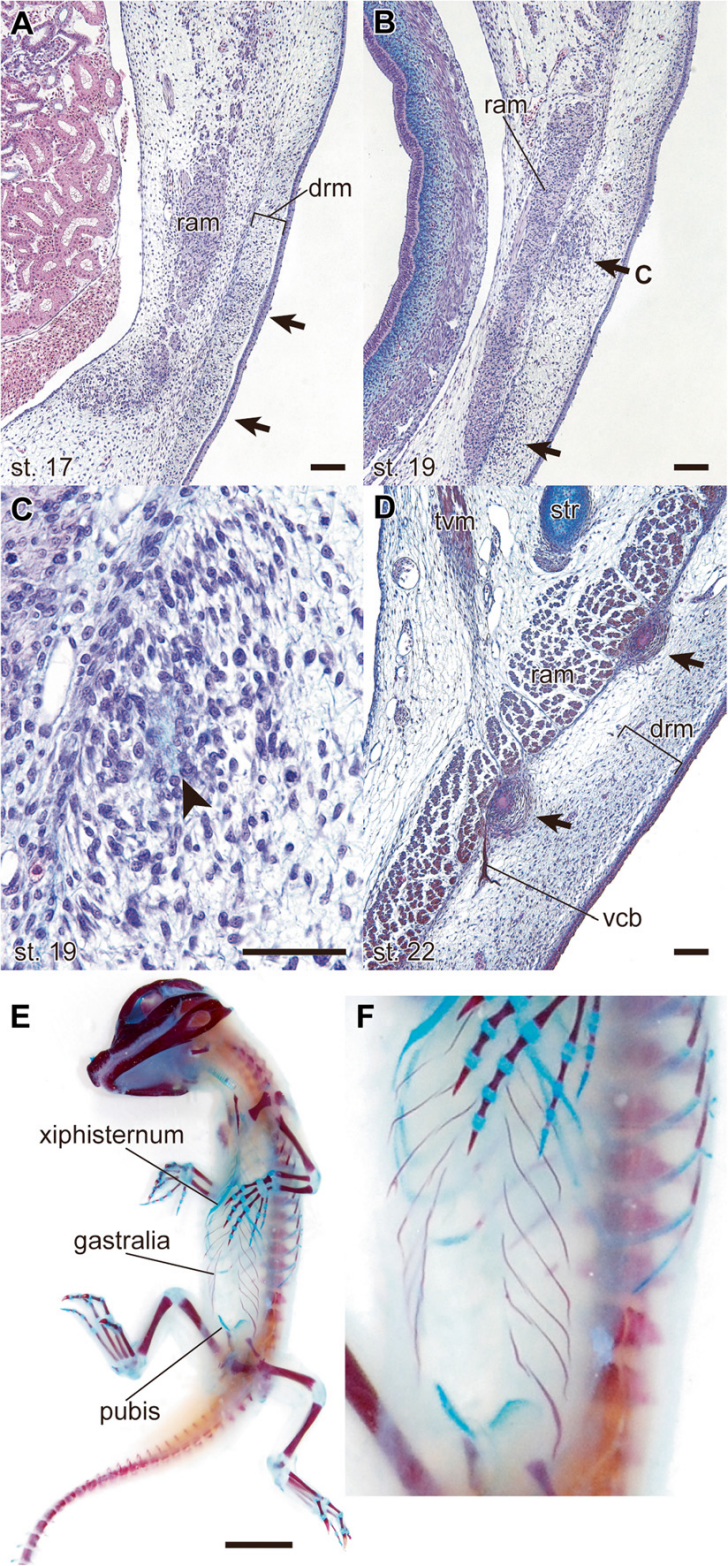
**Gastralia of the American alligator (** ***Alligator mississippiensis*** **).** The embryos were staged according to Ferguson (1985) [26]. **(A)** Transverse section of the ventral trunk of an embryo at stage 17. Formation of the gastralia begins with condensation of cells (arrows) in the dermis (drm). Alcian-blue, hematoxylin and eosin stains; scale bar, 100 μm. **(B)** Transverse section of the ventral trunk of an embryo at stage 19. The distance between the primordial gastralia and the rectus abdominis muscle (ram) decreases. Alcian-blue, hematoxylin and eosin stains; scale bar, 100 μm. **(C)** Enlarged image of the primordial gastralia, showing the matrix that is stained with Alcian blue (arrowhead), which appears transiently before the bony tissue is formed. Alcian-blue, hematoxylin and eosin stains; scale bar, 50 μm. **(D)** Transverse section of the ventral trunk of an embryo at stage 22. The gastralia contact the rectus abdominis muscle. The ventral cutaneous branch of the intercostal nerve (vcb) runs adjacent to the margin of the gastralium. Alcian-blue, hematoxylin, eosin and immunohistochemistry with anti-acetylated tubulin antibody (T6793, Sigma-Aldrich) stains; scale bar, 100 μm. tvm, transversus ventralis muscle. **(E)** Ventral view of a stage 25 embryo. Alizarin red and Alcian blue stains; scale bar, 1 cm. **(F)** Enlarged image of **E**.

A possible intermediate condition between ancestral and sunken exoskeletons is represented by the gastralia (Figure 2). The gastralia are a series of segmental rod-like bones that cover the ventral aspect of the abdomen in crocodilians and the tuatara, among living forms. Based on fossil evidence, the gastralia are thought to have evolved from exoskeletal bony scales and thus are exoskeletal elements [21]. However, the gastralia embryonically develop in close association with the rectus abdominis muscle in a deep layer, whereas other trunk exoskeletal elements develop close to the epidermis [22,23] (see also Figure 2). Accordingly, Hay (1898) [22] distinguished the gastralia from other dermal bones and classified the gastralia as “fascia bone.” Such a concept had been inherited in the distinction between “epithecal” and “thecal” ossifications, as used by Völker (1913) [24] and Zangerl (1939) [25], which indicate outer and inner dermal layers of ossification, respectively.

The previously mentioned evolutionary shifts in the topographic positions of exoskeletal elements recalls the idea of Holmgren (1940) [27], who suggested that, in some cases, various exoskeletal elements evolved into endoskeleton as the result of a topographic shift (delamination theory). However, studies of comparative morphology provide no evidence of interchangeability between endo- and exoskeletons [7]; the two historical lines of endo- and exoskeletal systems are likely to have evolved quite independently from each other. It is true that, in some cases, exposed endo- and exoskeletal elements become fused into a single element during ontogeny, as seen in the ontogenetic fusion between endoskeletal costal plates and exoskeletal peripherals to form the carapace in turtles, and in the fusion between endoskeletal vertebrae and exoskeletal osteoderms to form a tail club in ankylosaurid dinosaurs [28]. However, the ossification centers maintain their separate entities, implying incompatibility between the endo- and exoskeletons. (Nevertheless, it is worth noting that a vestigial component of the cleithrum (exoskeletal element) on the scapula (endoskeletal element) in mammals has been suspected repeatedly [29,30]. This evolutionary change represents a “phylogenetic fusion” advocated by Patterson, 1977 [7]).

Some skeletal elements cannot always be traced back to the ancestral endo- or exoskeleton. There are some examples of newly acquired endo- or exoskeletons in various derived taxa. In special cases, bones are sometimes produced within musculotendinous tissues as neo-formations in specific taxa (e.g., the ossified tendon [31]; and sesamoid bones) or by pathologic ossification. Smith (1947) [32] called these bones “subdermal bones,” whereas Patterson (1977) [7] classified them as membrane bones and components of the endoskeleton (Table 1).

As another example of newly evolved endoskeletal bones, the baculum is a cartilage bone that was newly acquired in the lineage of eutherian mammals [33]. Likewise, non-eutherian mammals have epipubic bones, which were newly acquired in the more basal mammalian lineage and lost in the crown eutherians [34]. It remains uncertain whether the baculum evolved from the epipubic bone of non-eutherian mammals [35], but examples of the baculum and epipubic bone are suggestive of a novel cartilage bone (a component of the endoskeletal system) that was acquired as an autapomorphy of a specific clade.

In addition, novel exoskeletal elements have been acquired in specific clades. The predentary and rostral bones are examples of such exoskeletal elements [36,37]. Osteoderms (the bony plates covering body contours) occur recurrently throughout vertebrate evolution [38-40]. Although morphological traits are distributed intermittently along the phylogeny, osteoderms are considered to share a developmental basis (“latent homology” *sensu* [40]), perhaps illustrative of the historical continuity of these bony elements [39,40].

### Histogenesis: endochondral and intramembranous ossifications

In contrast to the distinction of exo- and endoskeletons, adjectives such as ‘endochondral’, ‘dermal’ and ‘intramembranous’ are used exclusively for histogenetic aspects of skeletal tissues, and primarily unrelated to skeletal morphological identities [11]. In many cases, endoskeletal bones develop in association with preexisting cartilage, whereas exoskeletal bones develop solely intramembranously. However, some endoskeletal bones develop solely intramembranously, without any association with cartilage (membrane bones: Table 1), and some exoskeletal bones are likewise associated with cartilage. Comparative morphology studies have shown that cartilaginously preformed bone in the ancestral endoskeleton became intramembranously developed bone in derived taxa (e.g., the orbitosphenoid of the Amphisbaenia [16]). In contrast, cartilage (secondary or adventitious cartilage) develops on the periphery of exoskeletal bones that develops intramembranously, late in ontogeny of derived clades [7,41]. Cartilage has even been identified in the exoskeletal armor of the trunk (placodont sauropterygians [42]). Therefore, histogenetic modes with respect to the association of cartilage are interchangeable throughout evolution, as once suggested by De Beer (1937) [43].

Cartilaginously preformed bone is produced through both intramembranous (perichondral) and endochondral ossification. In perichondral ossification, the typical mode for periosteal bone formation, osteoblasts are differentiated from the perichondrium/periosteum surrounding the cartilage and subsequently produce the osteoid inside the periosteum. In the development of the costal plate of the turtle carapace, the periosteum expanded outward; therefore, osteoblasts produce outgrowths of the periosteal bone collar, or bony trabeculae [12].

**Figure 3:**
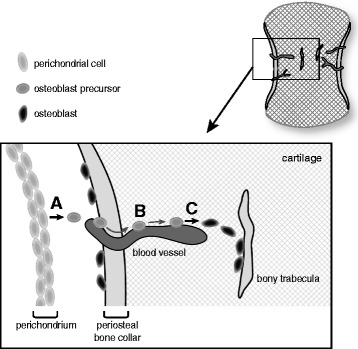
**Process of endochondral ossification. (A)** Differentiation of osteoblastic precursors from perichondrial cells. **(B)** Migration of osteoblastic precursors **(C)** Formation of bony trabeculae by mature osteoblasts.

Recent studies have shown that osteoblast cells derived from the perichondrium also support endochondral ossification [44]. In the early phase of this developmental process, osteoblastic precursors differentiate from perichondrial cells (Figure 3A) and subsequently migrate from surfaces in which the cartilage template is degraded into the primary ossification center of the endochondral bone (Figure 3B). Typically, blood vessels invade the cartilage from entrances of osteoblastic precursors and extend along their migration, suggesting intimate developmental relationship between vascularization and endochondral ossification [44]. The osteoblast precursors mature into osteoblasts to form bony trabeculae inside the cartilage (Figure 3C).

According to histological analyses of fossils, perichondral ossification evolved in the clade containing osteostracans and jawed vertebrates, whereas the endoskeletons of galeaspids comprise calcified cartilages, not perichondral bones [45]. Endochondral ossification originated evolutionarily in osteichthyes—that is, later than the emergence of perichondral ossification [46].

In the development of the cranial exoskeletal bones of extant osteichthyans, osteogenic cells are differentiated from mesenchymal condensations in the dermis. During this process of intramembranous ossification, osteoblasts mature from a specific transitional cell type (chondrocyte-like osteoblast), which co-expresses both osteogenic and chondrogenic marker genes [47].

Postcranial osteoderms (exoskeletal bones) develop in the dermis, presumably regulated by an intimate interaction with the epidermis. For example, in armadillos, the osteoderm is produced by osteoblasts that are differentiated from the condensation of dermal cells, with the orientation of the primordial osteoderm parallel to that of the epidermis [48]. In contrast, the osteoderm of alligators develops beneath the keel of scutes, but no osteoblasts are morphologically recognizable during this process [49]. There remains much room for investigation regarding the development of reptilian osteoderms.

In some fishes, exoskeletal bones are coated with enameloid or dentine tissues, namely, odontogenic components (reviewed by [50]). These enameloid- and dentine-coated bones occur widely among stem-osteichthyans, and odontogenic components are present in chondrichthyans also. However, the odontogenic components seen in chondrichthyans are believed to represent the vestige of the enameloid- and dentine-coated bones of ancestral jawed vertebrates, in which the bony portion was lost secondarily [51]—the exoskeleton of stem-gnathostomes likely was composed primarily of bone. This view is supported by recent histological data from placoderms (a taxon of stem-gnathostomes), indicating that the condition seen in extant chondrichthyans is derived. In placoderms, bony components always contributed to the exoskeleton, whereas odontogenic components did not always contribute to the exoskeleton [52,53], suggesting that odontogenic components were not prerequisite for exoskeletal development in these taxa.

In addition to endochondral and intramembranous ossifications there is a disparate mode of bone formation, namely metaplastic bone formation [54], the process by which preexisting tissues change directly (i.e., through metaplasia) into bony tissues. Exposed endoskeleton [12,55,56] and exoskeleton [57] contain portions of metaplastic bone, in which the collagen fibers of the dermis are engulfed.

**Figure 4:**
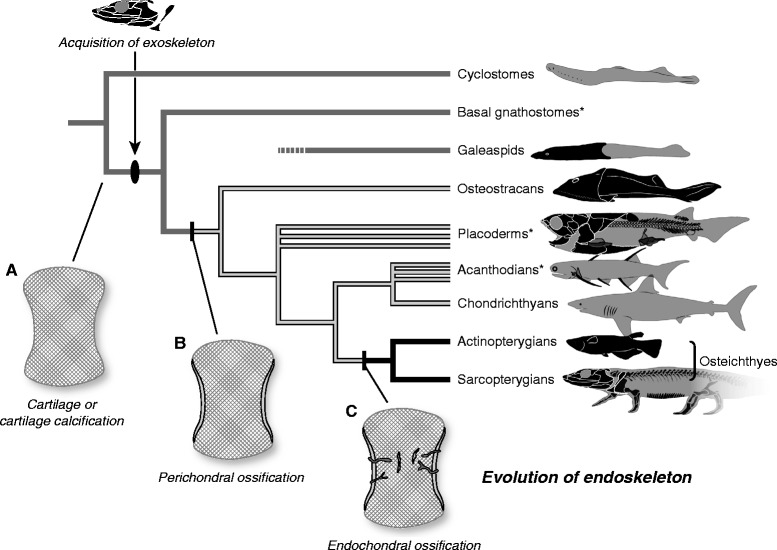
**Evolution of the endoskeleton.** Phylogenetic framework was adopted from [59]. Asterisks indicate paraphyletic groups. **(A)** Endoskeleton composed purely of cartilage. **(B)** Endoskeleton with perichondral ossification. **(C)** Endoskeleton with peri- and endochondral ossifications.

Collectively, comparisons of histogenesis in living and fossil vertebrates suggest the following scenario (Figure 4). In stem vertebrates basal to the clade of osteostracan-jawed vertebrates, the endoskeleton was composed purely of cartilage (Figure 4A). Osteostracans and non-osteichthyes jawed vertebrates evolved ossified endoskeletons (Figure 4B). In these animals, both endo- and exoskeletons developed purely through intramembranous ossification, although the endoskeleton developed on the surface of cartilage (perichondral ossification; as for perichondral ossification in chondrichthyes, see [58]). Osteichthyes acquired endochondral ossification, in which bony tissues are produced within (as well as on top of) cartilage (Figure 4C). During evolution, cartilage structures were occasionally lost and replaced in part by endoskeletal bones (membrane bones) and occasionally acquired in association with exoskeletal bones (secondary cartilages). Exoskeletal bones might be coated with enameloid and dentine tissues, but whether such a trait represents the ancestral or derived state is equivocal, on the sole basis of histological data. Alternatively, perhaps exoskeletal bones in the ancestral condition were not associated with enameloid and dentine tissues.

### Developmental origins and cell lineages—mesoderm and neural crest in the vertebrate cranium

Developmentally, the skeletal tissues of vertebrates have dual origins—the mesoderm and neural crest. Platt (1893) [60] suggested that the ectodermally derived mesenchyme (that is, ectomesenchyme) contributes to the cranial skeleton in basal vertebrates. De Beer (1958, 1971) [61,62] later used Platt’s notion to refute von Baer’s germ layer theory [63], because mesoderm generally was believed to be the main source of skeletal tissue in animals.

**Figure 5:**
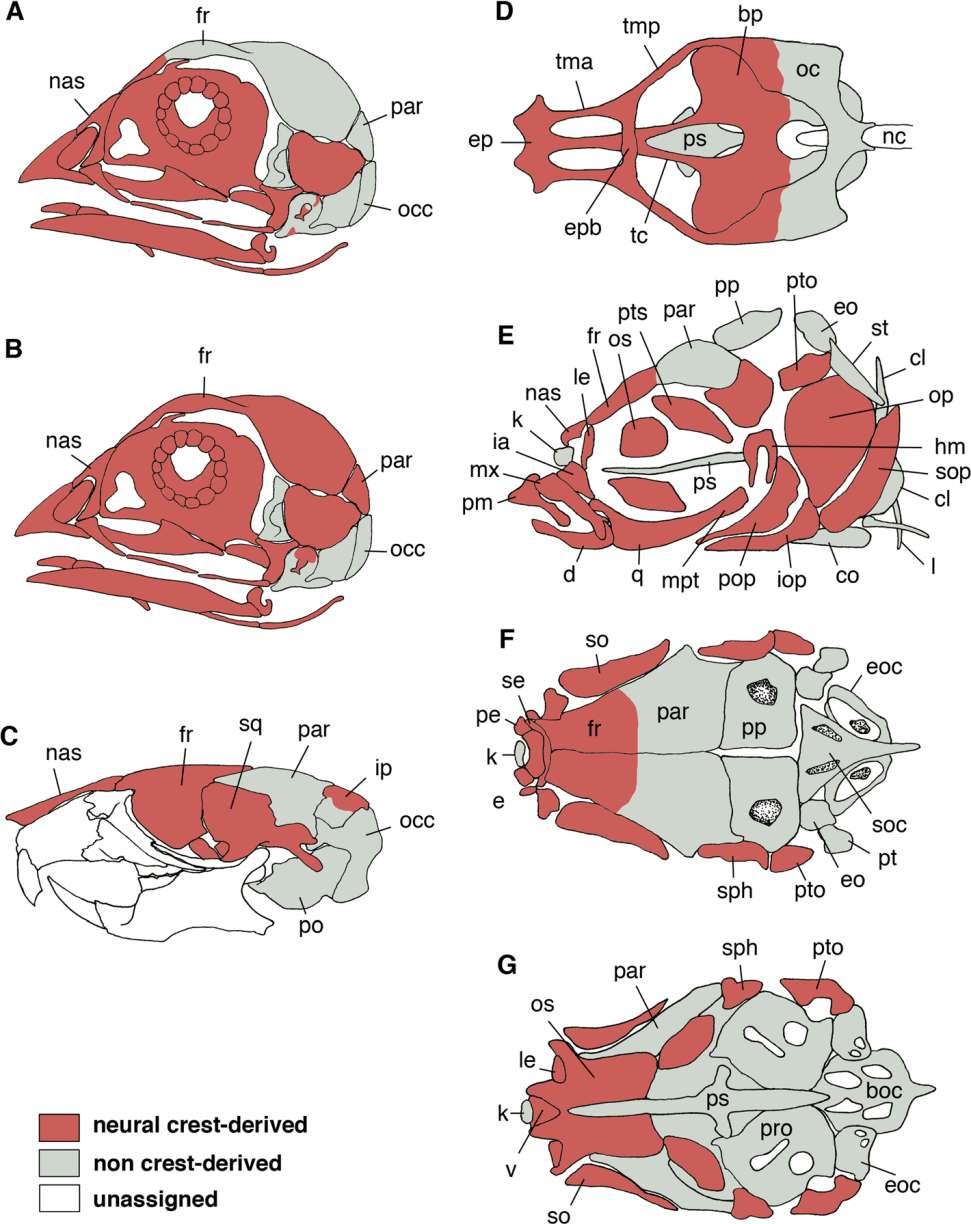
**Developmental origins of the dermal skull roof.** **(A** and **B)** Different views of the neural crest. Noden (1982, 1984) [80,81] placed the neural crest-mesodermal boudary in the dermal skull roof in the rostral part of the avian frontal **(A)**, whereas Couly at al. (1993) [82] reported that the entire dermal skull roof is derived from the neural crest. Note that the occipital represents an endoskeletal vertebral element secondarily assimilated to the cranium in gnathostomes. **(C)** Developmental origins of the dermal skull roof and the posterior cranium in the mouse, based on transgenic approaches by [70,72,83,84]. Neural crest-mesoderm boundary is located at the boundary between the frontal and parietal. **(D**-**G)** Neural crest- and mesodermal origins of the cranial elements in zebrafish based on transgenic techniques by Kague et al. (2012) [73]. Names of the bones were revised based on comparative osteology by [85,86]. Dorsal view of the chondrocranium **(D)**, and left lateral **(E)**, dorsal **(F)**, and ventral **(G)** views of adult zebrafish. In these views, the elements colored grey are of mesodermal origin. Note tha the neural crest-mesodermal boundary of the dermal skull roof is found in the frontal of this animal. Abbreviations: boc, basioccipital; bp, basal plate; cl, cleithrum; co, coracoid; d, dentary; e, ethmoid; eoc, exoccipital; fr, frontal; hm, hyomandibula; ia, intercalar; iop, interopercle; ip, interparietal; k, kinethomoid; le, lateral ethmoid; mpt, metapterygoid; mx, maxilla; nas, nasal; nc, notochord; oc, otic capsule; occ, occipital; op, opercle; os, orbitosphenoid; par, parietal; pe, preethmoid; pm, premaxilla; po, periotic; pop, preopercle; pp, postparietal; pro, prootic; ps, parasphenoid; pto, pterotic; pts, pterosphenoid; q, quadrate; se, supraethmoid; soc, supraoccipital; so, supraorbital; soc, supraoccipital; sop, subopercle; sph, sphenotic; sq, squamosal; st, supratemporal; tc, trabecula; tma, taenia marginalis anterior; tmp, taenia marginalis posterior; Redrawn from [8] **(A**-**C)** and from [73] **(D**-**G)**.

The origination of part of the vertebrate cranium from the neural crest has been exemplified through several experimental embryologic analyses involving amphibian and avian models in which neural crest grafting experiments are possible (reviewed by [64,65]). Even in non-model vertebrate species, including lampreys, similar results have been obtained [66,67] (also see [68,69]). The use of transgenic techniques has revealed the contribution of the neural crest to the skull in teleosts and mammals (Figure 5) [70-73]. It was previously thought that the rostral neural crest (cephalic crest) yielded mesenchymal tissue throughout the bodies of vertebrates, whereas the posterior portion (that is, the trunk crest) typically gave rise to a more limited repertoire of tissues, including melanocytes and the peripheral nervous system [74-77]. In the head, it has generally been accepted that the visceral arch skeleton (see below) is derived from the neural crest [78] (reviewed by [79]), which however, is not yet completely exemplified for some of the visceral dermal bones at the genetic level in the mouse (reviewed by [8]; Figure 5C).

In the context of comparative embryology and morphology, the cranium traditionally has been divided into several components, primarily the dorsal and ventral moieties (the neurocrania and viscerocrania, respectively) [43,79,87-94]. The neurocrania and viscerocrania are both recognized as endoskeletons over which a dermal covering, the dermatocranium, develops to encapsulate the entire endocranium. As noted earlier, the endocranium forms as a cartilage precursor and either ossifies through endochondral ossification to be replaced by bone, or degenerates (in cases in which dermal bones can perform the same functions). The cartilaginous skull roof in elasmobranchs is complete, but in animals in which the dermal skull roof is well developed that part of the cartilaginous neurocranium typically is absent.

Like the cranium, the dermatocranium can be divided into dorsal and ventral components corresponding to its neural and visceral elements. The cartilaginous neurocranium was initially recognized as a rostral continuation of the vertebral column, its elements being united and expanded to hold the enlarged brain. In contrast, the viscerocranium is composed of serial and metameric visceral arch skeletons surrounding the pharynx. In jawed vertebrates, one of the rostral elements is enlarged and divided dorsoventrally into the upper and lower jaws. The developmental origins of these cranial components have been, and remain, the focus of much debate.

According to Noden (1988)’s scheme [78], the neural crest-derived ectomesenchyme resides predominantly within the ventral part of the pharyngular head, in the region in which the craniofacial structures will form, whereas the majority of the cranial mesoderm is found more dorsally, lateral to the notochord and surrounding the brain primordium [78] (reviewed by [8]). This arrangement prompts the speculation that the distinction between neurocrania and viscerocrania will correspond to that of their embryonic cell lineages, i.e., mesoderm and neural crest. This seems reasonable, given that, like that of trunk somites, chondrification of the mesoderm is understood to require signals that emanate from the notochord. In contrast, the skeletogenesis of neural crest cells differs from that of the paraxial mesoderm, and is highly dependent on epithelial–mesenchymal interactions [82] (reviewed by [95]). Although this explanation holds true for part of the cranium, it is contradicted elsewhere.

**Figure 6:**
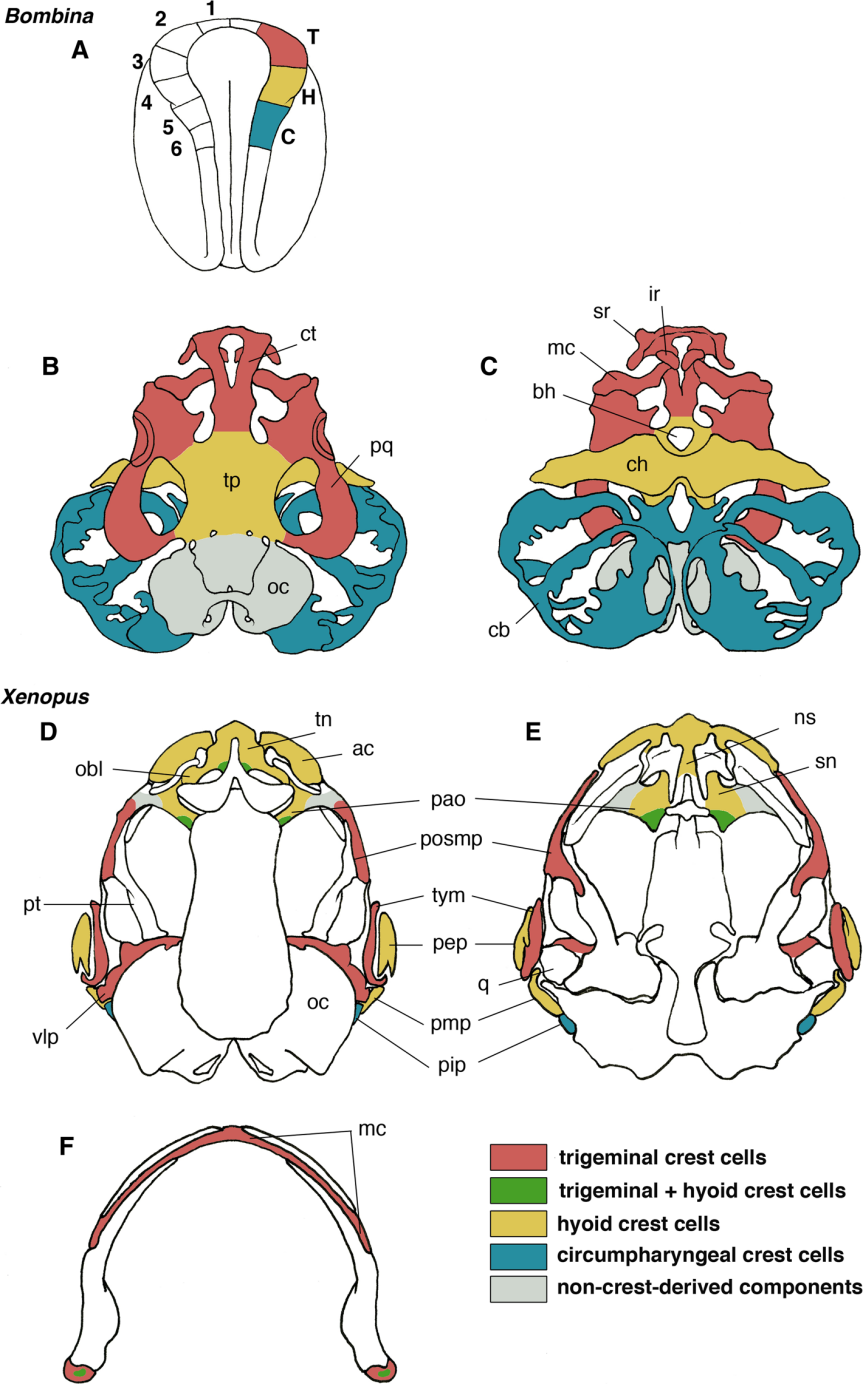
**Neural crest mapping of the anuran cranium.** **(A-** **C)** Mapping data in *Bombina orientalis* based on DiI injection onto the neural fold of the neurula **(A)**. From an experiment performed by Olsson and Hanken (1996) [110]. Origins and differentiation of three crest cell streams are colored in the right neural fold **(A)**, and dorsal **(B)** and ventral **(C)** views of larval chondrocranium. Trigeminal crest cells are colored red, hyoid crest cells yellow, and circumpharyngeal crest cells blue. Numbers on the left neural fold indicate sites of injections. Note that the trabecular plate (tp in **B**), generally derived from the premandibular crest cells, is mapped on the hyoid crest in *Bombina*. **(D-** **F)** Fate-mapping of adult *Xenopus* cranium. Dorsal **(D)**, ventral **(E)** views. Hyoid crest cells are distributed extensively in the sphenethmoidal region of the cranium. **(F)** Dorsal view of the lower jaw. Note that a part of the articular (proximal end of the Meckel’s cartilage) contains hyoid crest cells. Abbreviations: ac, alary cartilage; bh, basihyal; C, origin of circumpharyngeal crest cells; cb, ceratobranchials; ch, ceratohyal; ct, cornu trabecula; H, origin of hyoid crest cells; ir, infrarostral; mc, Meckel’s cartilage; ns, nasal septum; oc, otic capsule; obl, oblique cartilage; pao, planum antorbitale; pep, pars externa plectri; pip, pars interna plectri; pmp, pars media plectri; posmp, posterior maxillary process; pq, palatoquadrate; pt, pterygoid; q, quadrate; sn, solum nasi; sr, suprarostral; T, origin of trigeminal crest cells; tp, trabecular plate; tym, tympanic annulus; vlp, ventrolateral process. Redrawn from [111,112].

First, the so-called cranial base is not entirely made of mesodermal cells—its rostral portion (rostral to the position of hypophysis) is preformed as paired rods of cartilages called trabeculae, which are derived from the neural crest [71,82,96] (reviewed by [97]; Figures 5D, 6A–C). Comparative embryologists have suggested that this structure represents visceral arch skeletons that had been ancestrally developing rostral to the mandibular arch (reviewed by [97-99]). Although trabeculae in the cyclostomes are not homologous with those in jawed vertebrates, it is now generally accepted that the rostral part of the neurocranium originates from the neural crest throughout the vertebrate species [79,100,101] (also see [68,102]). Therefore, in a developmental sense, the endoskeletal neurocranium is a composite structure, derived from both the mesoderm and cephalic neural crest. Its posterior part, which originates mainly from a pair of longitudinal plates called parachordals, is a mesodermal structure, except for the otic capsule, which is derived partly from the neural crest. The parachordals secondarily incorporate segmented somitic (vertebrae-like) materials to complete the posteriormost portion, the occipital region [43,90,103-106]. In the chicken, and in other sauropsids as well, this part of the neurocranium contains five somites [43,82]. Therefore, as far as this portion is concerned, the vertebrate cranium—like the vertebral column—is segmented, as suggested by transcendental morphologists [2,107,108] (also see [109]).

**Figure 7:**
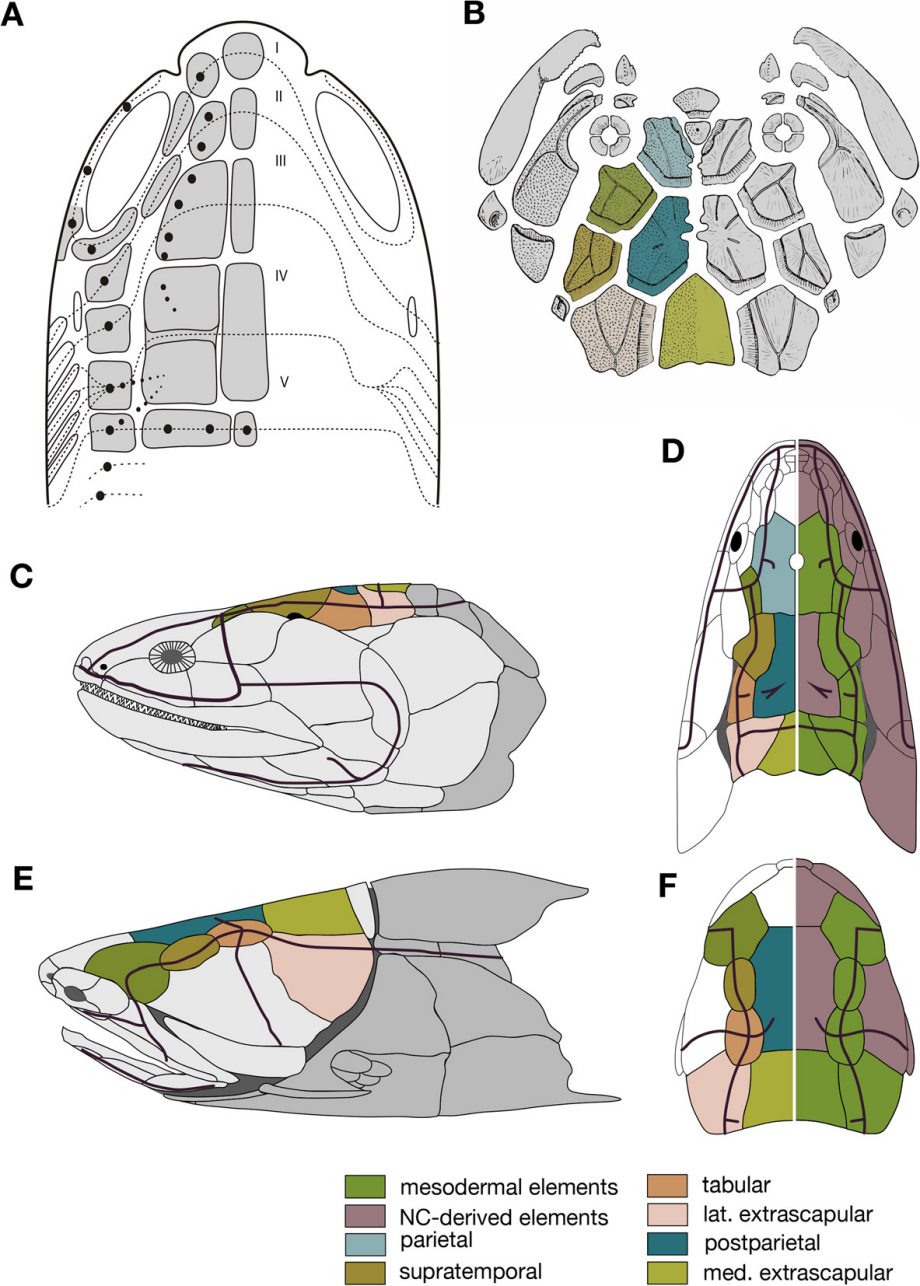
**Evolution of dermatocranial elements.** **(A)** Traditional scheme of the dermal skull roof, based on the head segmentation scheme of Jollie (1981) [113]. **(B)** Schematized prototype of the arthrodire dermal skull roof as suggested by Heintz (1932) [115]. Homologies between various dermal elements in **B** and **F** are indicated by color. **(C-F)** Dermatocranium of *Eustenopteron* **(C** and **D)** and *Entelognathus* **(E** and **F)**, lateral **(C** and **E)** and dorsal **(D** and **F)** views. Thick red lines represent lateral lines that correspond to patterns of some dermal elements. Presumed homologous dermal elements are shown in the same color in **C** and **E** and the left halves of **D** and **F**. On the right side of **D** and **F**, neural crest- and mesoderm-derived elements are differently colored according to assumptions that the crest–mesoderm interface is primarily found between the frontal and parietal bones (as in the mouse) and that postparietal homologues are consistently derived from the neural crest in sarcopterygians (including tetrapods). **C-F**, redrawn from [59].

The developmental origins of the dermatocranium are more enigmatic, creating an obstacle to the understanding of its evolution, and vice versa (Figures 5, 6 and 7). According to classical theory, transcendental morphologists and others believed that the anteroposterior segmentation of the roof of the dermatocranium merely reflected the pattern of cranial mesodermal segments of hypothetical ancestors (reviewed by [92,113,114]; Figure 7A). However, this conventional assumption, which was captured through morphological comparisons (before evolution was conceptualized), is incompatible with our current understanding of developmental origins. Again—in all vertebrate embryos examined so far—the neural crest contributes to both the visceral part of the calvarium and the neural components of the dermatocranium.

The dermal elements of the calvarium are likely patterned according to the lateral line system, and thus the homology of these elements is, in aquatic forms, based on the homology of lateral lines (see [59,114] and references therein; Figure 7C–F). Although the patterns of dermal bones and lateral lines are coupled developmentally, it is unclear whether the lateral line induces the dermal bones, or vice versa (see [116]). Presumably the typical dermal bones found in fishes (including placoderms) became secondarily sunken exoskeletal elements concomitant with the shift in developmental interactions to induce membranous ossification in a deeper layer of the dermis, as found in amniotes. Questions remain regarding homologies (evolutionary continuities) of the dermal elements (reviewed by [8]), as well as their early evolution. The pattern of the dermal skull roof perhaps was first established in placoderms [59] (Figure 7B–F; also see [115]), in which the topographic relationship between dermal bones and lateral lines seen in modern vertebrates is recognizable, at least in part. From lines of circumstantial evidence regarding neural crest contribution and its putative relationship with lateral lines, it is unlikely that the dermal skull roof elements represent segmental organization of the vertebrate head. The lateral lines are not induced as primordia with any segmental prepatterning (for the developmental pattern of the placodes, see [117] and references therein); therefore, the dermal skull roof elements may form independently of any segmental prepattern.

By constructing chick–quail chimeras, Noden found that the rostral part of the dermal skull roof is derived from the neural crest, whereas the posterior arises from the mesoderm [80,81,118,119] (Figure 5A). The boundary between these two cell lineages lies in the frontal bone (for the homology of the avian frontal bone, see [8]). Similar results from a similar experiment were obtained by Le Lièvre (1978) [120]. However, Couly et al. (1993) [82] showed that the entire dermis, as well as the dermatocranial elements, is exclusively of neural crest origin (Figure 5B). To date, systematic fate mapping of the avian craniofacial structures has not been completed; the explanation underlying these inconsistent results remains unclear, but may involve contamination by non-crest tissues or incomplete postsurgical wound healing (summarized by [8]).

Regardless, the views of Couly et al. (1993) [82] once prevailed among zoologists and carried the expectation that the entire exoskeleton of vertebrates—head and trunk—would be of neural crest origin (reviewed by [121]). Another finding that appeared to strengthen this assumption was that the differentiation repertoire of the neural crest is not entirely predetermined differentially along the anteroposterior axis (head versus trunk); heterotopically transplanted trunk neural crest can exhibit skeletogenic potency in the head environment of the embryo [122] (also see [123] for a similar experiment; also see [124]). It was thus speculated that the trunk neural crest is normally suppressed from differentiating into the exoskeleton in animals that have lost most of the postcranial exoskeleton, which, however, can be reactivated under specific circumstances. In fact, all exoskeletal elements in vertebrates, including the dermal skull roof, teleost scales, lepidotrichia, and the extensive head shield in some fossil lineages such as osteostracans and placoderms, were expected to originate from the neural crest [17]—despite the lack of any supporting evidence for this notion. This overly simplified prediction was further extended to postulate the involvement of the neural crest in the turtle shell, which had often been interpreted erroneously as an exoskeletal element (see [12]; see above). Here, the mesoderm-crest duality was related to an in–out topography of endo/exoskeletal parts in the neurocranium, not along the dorsoventral axis.

New embryonic technologies have apparently dispelled the above unsubstantiated assumptions. Shimada et al. (2013) [77], for example, performed transplantations of somites and neural crest in medaka embryos and convincingly showed that the trunk scales of this fish originate from the mesoderm, not the neural crest. Analyses of transgenic lines of zebrafish by several other groups yielded similar results [75,125]. However, several groups suspect that the neural crest contributes to the exoskeleton of the trunk, for example, to the lepidotrichia of the caudal and dorsal fins in zebrafishes [73] and the turtle plastron [124,126]. Furthermore, a recent study tracing the lineages of transgenic cells revealed that trunk neural crest cells do not generate a skeletogenic tissue (that is, ectomesenchyme) [76] although they have skeletogenic potential in the developing head [122]. These lines of evidence, in combination with the fossil evidence from placoderms [52,53] (see above), suggest that the exoskeleton of the trunk develops from the mesoderm in the ancestral condition in the jawed vertebrates and that accretions of the enameloid and dentine tissues (i.e., odontogenic component) to the trunk exoskeleton occurred in many lineages, distinct from what had previously been hypothesized (e.g., [127]).

Consequently, the interface between the neural crest- and mesoderm-derived parts of the exoskeleton again appears to be somewhere in the skull roof, and different results regarding its specific location have been obtained via different experimental methods in embryos of different taxa (reviewed by [8]; Figure 5A, B). Our current understanding regarding the origin of vertebrate skull roof is therefore confused.

Transgenic technology was used to label crest-derived ectomesenchyme and its derivatives in mice (Figure 5C) [30,70,72,83]. In this model, the *Wnt-1* promoter was used to drive *Cre* to activate a reporter gene as a marker for all neural crest cells. This methodology resulted in labeling of the nasal, frontal, and interparietal regions in addition to the more ventrally located dermal elements, and the signal distribution was complementary to the pattern obtained by using *Mesp1*-*Cre*/R26R to label mesodermal cell lineages (see [72]). This result resembles those of Noden (1978, 1982, 1983, 1984) [80,81,118,128] and Le Lièvre (1978) [120] in avian embryos (Figure 5A; Evans and Noden, 2006 [119], subsequently confirmed these previous results by labeling mesoderm through retroviral infection). Furthermore, these current and previous findings coincide perfectly if we admit misidentification of the boundary between the frontal and parietal regions in mammals and avians: the supraoccipital region is the dorsal portion of a mesodermal element serially homologous with the vertebrae, and the interparietal region may not be present in avians (for the homology and evolution of the interparietal region, see [129] and references therein).

One consistent aspect in this conundrum is that every argument has been based on the firm assumption that evolutionarily conserved bony elements should arise from fixed (homologous) cell lineages in development. This assumption is, of course, profoundly linked to the cell-autonomous and precommitted potency of the neural crest cells in morphological skeletal patterning (see [118,130-133]), which is not per se completely correct [128,134]. Accordingly, the comparative morphological understanding cannot easily be formulated into a simple developmental scheme [8]; in particular, developmental understanding of the neural crest–mesodermal boundaries in the dermatocranial roof is conspicuously unsure compared with that for the cranial base. Several evolutionary scenarios, not always mutually exclusive, may explain the situation regarding the origins of the dermatocranial roof:

1. Morphological homologies of bony elements and the cell lineages that give rise to these elements are regulated at different, decoupled levels, and the bony elements can be conserved through evolution independent from the cell lineages, which are apt to change more rapidly.
2. The ancestral developmental pattern and cell-lineage origins of the dermatocranial elements were established in various fossil taxa, which are reflected in some modern taxa, and are secondarily modified in others, possibly because of the loss or fusion of ancestral elements or the addition of new elements.
3. The dermatocranium (excluding the supraoccipital region) primarily was derived from the cranial neural crest ancestrally, and new mesodermal elements intercalated secondarily to accommodate adaptation to the expansion of the cranial vault in different ways in each animal lineage, obliterating homologies between some bones (as suggested in Figure 7, the parietal bone represents a newly inserted mesodermal element).
4. The dermatocranium (excluding the supraoccipital bone) was primarily derived from the mesoderm ancestrally, and new crest-derived elements were intercalated secondarily to accommodate adaptation to the expansion of the cranial vault in different ways in each animal lineage, thus obliterating homologies of bones.
5. The pattern of dermal elements belongs to most variable parts of the vertebrate body, and developmental constraints assure homologies of dermal elements only within limited levels of taxa (orders, superfamilies, etc.; see [135-137]; reviewed by [113]).
6. Mesodermal dermal elements were associated primarily with various lateral lines in ancestral forms, and other elements were all derived from the neural crest (Figure 5D and F).
7. The lateral line-induced dermal elements in ancestors have been lost, and the tetrapod dermatocranium, predominantly derived from the neural crest, has been newly reorganized in each animal lineage in its unique way.

None of the above scenarios has been assessed experimentally to date, nor have discrepancies among experimental embryologic data been reconciled. According to the third scenario, the parietal would have to be regarded as a synapomorphy in crown gnathostomes, which however, may be refuted by the fact that the majority of placoderms possess this bone [59].

The situation may be even more confusing than that presented. If the apparent inconsistency in the mesoderm–neural crest boundary could be explained, it may turn out to be attributable to a misnaming of bony elements; this could be resolved by morphological and developmental reexamination of homologous relationships [111]. Unfortunately, however, this confusion may be destined to be insurmountable. Transgenic and chimeric approaches have revealed that the cranium of the frog violates generally accepted rules of development—that is, the developmental origins of the visceral arch and craniofacial skeletons are not found in a canonical set of crest cell streams that are divided into mandibular, hyoid, and branchial arch streams; instead, morphologically homologous dermal elements are derived from inconsistent cell lineages in frog embryos (Figure 6D-F) [111,138]. Therefore, the skeletal development of the frog demonstrates the decoupling of embryonic patterns, cell lineages, and adult morphology in a very radical manner. It is conceivable that, especially in animals that go through metamorphosis, insertion of larval stages causes topographical shifts of the neural crest-derived chondrogenic cells that go on to form adult skeletons (although this does not explain the hyoid crest-origin of the prechordal cranium in amphibians as reported by Olsson and Hanken (1996) [110]). The same may be the case in the development of the dermal skull roof; the morphological patterns and homologies may reside in the local environment of the embryos, such that they become specified during a later phase of development. This potential influence of the local environment recalls the study of Schneider (1999) [139], in which cranial neural crest from the quail embryo was ectopically grafted within mesenchymal populations destined to form the skull wall in the chicken embryo. In resulting chimeras, these grafted cells gave rise to a skeletal element, which in birds is normally derived from the mesoderm. There are many more examples that demonstrate the importance of local tissue interaction in the specification of bony elements [128,140] (also see a review by [92]) by showing potential shifts of cell lineages and populations to generate morphologically conserved skeletal patterns during evolution.

A similar situation is seen in the apparent discrepancy of the neural crest contributions to the pectoral girdle bones between amniotes [30,141] and anamniotes [73,142]. It is generally accepted that, within the mesodermal cell population, the developmental basis providing the skeletal identities of the digits shifted between non-homologous primordia in the evolution from dinosaurs to birds (frame-shift hypothesis) [143-145]. No accounts contradict the possibility that skeletal identities similarly shift between neural crest and mesodermal cell populations.

### Perspectives—beyond the complexity

The vertebrate skull initially attracted the attention of zoologists because of its complex and elegant morphology, but its complexity clearly exceeds all expectations. Theories regarding skeletogenesis and skeletal anatomy and its evolution have been—and still are—fraught with confusion, which never seems to be resolved easily. This situation cannot be ascribed only to the misuse of terminology in non-comparable contexts of discussion; it also reflects the complexity of the developmental and evolutionary diversity of the vertebrate skeletal system per se. Nor is the current developmental understanding of skeletogenesis formulated in an orderly way into the pattern of embryos and cell lineages.

The dilemma described here is tightly linked to the confusion regarding the concept of homology. As noted earlier, morphological homology was in the past reduced to its developmental origins in cell lineages and germ layers, as seen in von Baer’s germ layer theory (reviewed by [6]). This theory was refuted as being based on inaccurate concepts of histogenesis, including the concept that skeletogenic differentiation can take place equally in mesodermal and ectodermal (neural crest) cell lineages. Still, the neural crest – mesoderm distinction, as well as endochondral–membranous ossifications, was expected to coincide with specific morphological components of the skull—a belief that could be viewed as a modified version of the germ layer theory. Alternatively, a similar reductionist argument was once widespread with a vague expectation in the dawn of evolutionary developmental biology; namely, that morphologically homologous structures should be patterned through certain unchanged infrastructures, like function of evolutionarily conserved sets of regulatory genes or gene regulatory networks.

Expectations such as these often come true, as typically exemplified by the isomorphic shifts of vertebral formula and Hox code [146] (also see [147]). In this context, the positional identities of vertebrae along the anteroposterior axis of the vertebral column (such as occipital, cervical, thoracic, lumbar, and sacral in mammals) coincide precisely with the expression domains of Hox genes in the prevertebral anlagen, and under this Hox-code-mediated specification the number of segments can vary during evolution (for variable numbers of vertebrae, see [147]). In this case, morphological homology is reduced to the regulation of homologous Hox genes. Similar situations, in which the homology between structure and gene expression is tightly conserved, include the expression of homeobox genes and primordial segments in the developing vertebrate brain, differentiation of somite-derivatives, and dorsoventral specification of the neural tube (reviewed by [148]). In an extreme reductionist argument that is focused on genes, cell-type identities, which are classified by transcript repertoire (that is, molecular fingerprinting of cell types), are comparable among phyla, even between the vertebrate- and annelid body plans, for example, at the level of single neurons [149].

Unfortunately, relationships among homologies at different hierarchal levels—namely at the levels of morphology, histogenesis, cell lineage and genes—remain murky, as homologous skeletal elements can arise from different or shifted cell lineages throughout evolution by means of different mechanisms of development, thus challenging the criteria for morphological homology (e.g., [5,150,151]; reviewed by [152]). Inconsistency of this type occurs in various phenomena of organogenesis, in which homologous structures are patterned by the actions of non-homologous regulatory genes in each animal lineage [153,154]. In the evolutionary context, there are at least two significant effects worth considering.

One effect is evolutionary novelty and simultaneous loss of homology: the shift in developmental interactions in time and place result in novel regulation of skeletogenic genes, leading to a skeletal pattern incomparable to that in the ancestor. The other effect is developmental drift: the developmental process and mechanisms would shift without changing the readout of the shifted developmental process, thus maintaining the ancestral morphological pattern in the adult. De Beer (1958) [61] noted the heterochronic factor behind similar phenomena, for example, in the creation of the larval stage in development. One of the most conspicuous examples is found in the columella auris (that is, hyomandibular bone) of certain frogs. In *Xenopus*, the anlage of the columella never appears during the larval stage, but arises during metamorphosis [155,156]. In the mouse, the stapes (the homologue of this cartilage bone) is patterned during embryogenesis in the dorsal part of the second pharyngeal arch and is specified through the upregulation of *Hoxa2* [131] in the ectomesenchyme. In *Xenopus*, homology of this skeletal element appears to be maintained—albeit decoupled from the Hox code— and its differentiation is even suggestive of new involvement of the thyroid hormone in the rewired regulatory network. We have already seen, in frog development, how morphologically homologous cranial elements arise from cell populations or pharyngeal arches not identical to those in other vertebrate groups.

It is true that the morphological homology of skeletal elements cannot be reduced directly to the developmental program, or homology of genes, involved in the generation of homologous structures. However, insofar as the criteria for homology largely rest on the relative positions of organs (reviewed by [6]), developmental patterns may, to some extent, explain the impetus behind the manifestation of the homologous patterns. This explanation is especially plausible given that the relative positions suggest evolutionarily maintained topography of cell populations and tissues, which act as the bases for embryonic interactions to establish the identities of the skeletal anlagen, especially through the upregulation of specific sets of transcription factor-encoding genes. Here we recall the experiment of Schneider (1999) [139] to show that neural crest-derived ectomesenchyme and cephalic mesoderm can be exchanged to generate morphologically normal chondrocranium. This experiment indicates that the developmental factor(s) for the morphological homology resides in the “position” in the embryo, not in the embryonic cell lineages. Consistently, a same set of gene expressions has been detected in endochondral ossifications of mesenchymal condensations both derived from neural crest and mesodermal cells [157]. This implication stands in conspicuous contrast to the fact that species-specific shape appears to evolve in the developmental program associated with specific cell lineages [130,133].

To understand the mechanistic background for the burden of development, we have to understand how selective pressure—especially stabilizing selection—at the phenotypic level (adaptation) acts on the developmental program exerted from the genome. In other words, we must identify parts or elements of the developmental program (for example, gene regulatory networks, modules, sets of regulatory genes and their regulatory elements) that can or cannot change when certain fixed phenotypic patterns are favored. These efforts will uncover the aspects of the developmental program that are resistant to change and those that are apt to change during evolution. In evolution, adaptation and constraint cannot be discriminated a priori [158,159]. The key to discriminating between these two causal relationships behind evolution is provided abductively through historical and experimental analyses of the correlation between phenotype and the developmental program behind it (for example, skeletal elements can be considered as a phenotype of a skeletal system). The patterns that allow minimal shifts have been recognized to result from developmental constraint. The concept of developmental constraint has not yet been explained thoroughly, but taxon-specific conserved patterns of embryogenesis have been recognized as the so-called “phylotype,” which tends to appear in the organogenetic stage of development (“phylotypic stage” [160]).

In transcendental morphology, the phylotype (pharyngula in vertebrates) has been viewed as an embodiment of the conceptual archetype, a shared morphology of the embryos of animals belonging to the vertebrates, from which various types of adult morphologies can be derived [63]. This “derivation,” however, does not necessarily refer to the phylogenetic evolutionary process, but rather to observers’ perceptions of homologous patterns and their developmental changes. Morphologically, it is true that the pharyngula-stage embryo in vertebrates is the stage at which the basic body plan, or a set of homologous anlagen, of this animal group becomes established. In the evo-devo context, the phylotypic stage of vertebrate development is recognized as the stage at which so-called tool-kit genes (typically the Hox code) are expressed most conspicuously during development, thus providing the mechanistic bases to explain the significance of this conserved embryonic pattern [160]. Recent transcriptome analyses have shown that the most similar gene expression profiles coincide with the phylotypic stage [161]. Importantly, as indicated by genomic analyses of turtles, the evolutionarily novel patterns of the skeletal system in vertebrates appear to arise through spatiotemporal developmental shifts after the establishment of the above-noted phylotype [162]. Taking into consideration the shifts in morphological homologies—specifically the developmental patterns and processes involved in patterning of the evolutionarily fixed patterns of craniofacial elements—it seems likely that the cranial pattern is specified late relative to the specification of the phylotype. This delay suggests the presence of another developmental constraint, which is more or less uncoupled from those needed to maintain the phylotype. The search for such taxon-specific constraints, as well as their mechanistic importance, is an intriguing focus for future evo-devo studies. The results likely would further our understanding of the synapomorphies used in the reconstruction of evolutionary history.
